# Incorporating physiological knowledge into correlative species distribution models minimizes bias introduced by the choice of calibration area

**DOI:** 10.1007/s42995-024-00226-0

**Published:** 2024-05-13

**Authors:** Zhixin Zhang, Jinxin Zhou, Jorge García Molinos, Stefano Mammola, Ákos Bede-Fazekas, Xiao Feng, Daisuke Kitazawa, Jorge Assis, Tianlong Qiu, Qiang Lin

**Affiliations:** 1 CAS Key Laboratory of Tropical Marine Bio-resources and Ecology, South China Sea Institute of Oceanology, Chinese Academy of Sciences, Guangzhou, 510301 China; 2 Guangdong Provincial Key Laboratory of Applied Marine Biology, South China Sea Institute of Oceanology, Chinese Academy of Sciences, Guangzhou, 510301 China; 3 Marine Biodiversity and Ecological Evolution Research Center, South China Sea Institute of Oceanology, Guangzhou, 510301 China; 4 Global Ocean and Climate Research Center, South China Sea Institute of Oceanology, Guangzhou, 510301 China; 5 Institute of Industrial Science, The University of Tokyo, 5-1-5 Kashiwanoha, Kashiwa, Chiba 277-8574 Japan; 6 Arctic Research Center, Hokkaido University, Sapporo, Hokkaido 001-0021 Japan; 7 Finnish Museum of Natural History, University of Helsinki, Helsinki, Finland; 8 Molecular Ecology Group (MEG), Water Research Institute (IRSA), National Research Council of Italy (CNR), 28922 Verbania Pallanza, Italy; 9 National Biodiversity Future Center (NBFC), Palermo, Italy; 10 Institute of Ecology and Botany, HUN-REN Centre for Ecological Research, Vácrátót, Hungary; 11 Department of Environmental and Landscape Geography, ELTE Eötvös Loránd University, Budapest, Hungary; 12 Department of Biology, University of North Carolina, Chapel Hill, NC 27599 USA; 13 Centre of Marine Sciences, University of Algarve, Campus de Gambelas, Faro, Portugal; 14 CAS Key Laboratory of Experimental Marine Biology, Institute of Oceanology, Chinese Academy of Sciences, Qingdao, 266071 China; 15 University of Chinese Academy of Sciences, Beijing, 100049 China

**Keywords:** Bayesian approach, Climate change, Habitat suitability, Physiological knowledge, Species distribution model

## Abstract

Correlative species distribution models (SDMs) are important tools to estimate species’ geographic distribution across space and time, but their reliability heavily relies on the availability and quality of occurrence data. Estimations can be biased when occurrences do not fully represent the environmental requirement of a species. We tested to what extent species’ physiological knowledge might influence SDM estimations. Focusing on the Japanese sea cucumber *Apostichopus japonicus* within the coastal ocean of East Asia, we compiled a comprehensive dataset of occurrence records. We then explored the importance of incorporating physiological knowledge into SDMs by calibrating two types of correlative SDMs: a naïve model that solely depends on environmental correlates, and a physiologically informed model that further incorporates physiological information as priors. We further tested the models’ sensitivity to calibration area choices by fitting them with different buffered areas around known presences. Compared with naïve models, the physiologically informed models successfully captured the negative influence of high temperature on *A. japonicus* and were less sensitive to the choice of calibration area. The naïve models resulted in more optimistic prediction of the changes of potential distributions under climate change (i.e., larger range expansion and less contraction) than the physiologically informed models. Our findings highlight benefits from incorporating physiological information into correlative SDMs, namely mitigating the uncertainties associated with the choice of calibration area. Given these promising features, we encourage future SDM studies to consider species physiological information where available.

**Supplementary Information:**

The online version contains supplementary material available at 10.1007/s42995-024-00226-0.

## Introduction

Climate change affects all ecosystems on Earth—terrestrial (Chen et al. 2011; Mammola et al. 2019a), freshwater (Woodward et al. 2010), and marine alike (Brito-Morales et al. 2020; Levin and Le Bris 2015)—being recognized as one of the five direct drivers responsible for global biodiversity loss (IPBES 2019). As the largest ecosystems on Earth, oceans are now experiencing dramatic changes due to multiple drivers, such as warming, acidification, and deoxygenation (Doney et al. 2012; Poloczanska et al. 2013). These changes have been reported to adversely affect organisms via diverse ecological mechanisms (Doney et al. 2012; Poloczanska et al. 2013, 2016), of which geographical range shifts of species are one of the most frequently observed outcomes (Doney et al. 2012; Dong et al. 2024; Lenoir et al. 2020; Pinsky et al. 2020). In response to climatic warming, marine organisms are shifting either poleward (Lenoir et al. 2020; Pinsky et al. 2020) or to deeper waters (Dulvy et al. 2008; Pinsky et al. 2013) in search of cooler environments. In a recent global study, Lenoir et al. (2020) demonstrated that, on average, marine species have shifted poleward over the course of the last century six times faster than species on land (5.92 versus 1.11 km per year, respectively). This quick global rearrangement of the mosaic of marine biodiversity can alter the composition and profitability of global fisheries catch (Cheung et al. 2013; Gaines et al. 2018) and affect population stability by creating novel species interactions (Potts et al. 2014; Vergés et al. 2016). For implementing effective management measures, it is critical to obtain a nuanced understanding of how species may respond to future scenarios of climate change.

Correlative species distribution models (SDMs) are tools to estimate species’ habitat suitability by describing the statistical relationships between species distribution data and relevant predictors, which have become popular to investigate species response to climate change (Araújo et al. 2019; Feng et al. 2019; Guisan et al. 2017; Qiao et al. 2015; Taheri et al. 2021). Despite their growing popularity, correlative SDMs depend heavily on the quality of species distribution data, which are often susceptible to sampling bias and spatial uncertainty (Araújo et al. 2019; Feng et al. 2019; Hughes et al. 2021; Kramer‐Schadt et al. 2013; Marcer et al. 2021). Furthermore, among the recognized limitations of correlative SDMs (e.g., Ryo et al. 2021; Smith et al. 2019; Zhang et al. 2024), their inability to account for species physiological limits is a major drawback (Hof 2021; Kearney and Porter 2009; Liao et al. 2021). Specific environmental preferences and tolerances mean that, although species may occupy a broad spatial extent, the spatial variability in the suitability of those habitats could be large (e.g., Champion et al. 2020). In other words, the habitat suitability of species occurrence records is not equal: some occurrences are from species optimal habitats, whereas others may be collected from marginally suitable regions. Given these biases in species distribution data, correlative SDMs without considering species’ physiological knowledge are at high risk of failing to properly capture the environmental requirements of target species.

Compared with correlative SDMs, mechanistic SDMs directly use the physiological requirements of species to reasonably describe the realized niche (Dong et al. 2024; Kearney and Porter 2009; Peterson et al. 2015). However, mechanistic models require a number of physiological measurements for proper parameterization. These data are often costly and time-consuming to acquire, limiting the wide application of these models (Mammola et al. 2021; Peterson et al. 2015). Given the growing availability of physiological information, in addition to using species’ physiological constraints to guide the selection of appropriate biologically relevant predictors, it is timely to explore the possibility of incorporating physiological knowledge directly into the fitting of correlative SDMs. In this regard, Bayesian SDMs, which are based on Bayes’ theorem (Bayes 1764), make statistical inferences by incorporating species’ physiological information as prior knowledge. Thus far, different Bayesian approaches have been developed and successfully applied in various studies, improving SDM performance and producing more reliable results (e.g., Brewer et al. 2016; Golding and Purse 2016; Talluto et al. 2016; Zhu et al. 2021). For example, Feng et al. (2020) tested the importance of integrating physiological knowledge into SDMs for zebra mussel *Dreissena polymorpha* (Bivalvia: Dreissenidae) and reported that physiologically informed models improve model extrapolation ability. Similarly, Gamliel et al. (2020) compared the performance of physiologically informed and naïve SDMs for six Mediterranean marine species, demonstrating that models incorporating physiological information project less extreme range shifts. Despite the promising potential of integrating different data sources (i.e., distribution data and physiological knowledge), the significance of physiologically informed SDMs has not been fully appreciated yet, and the applications of incorporating physiological information into SDMs are still limited (e.g., Mammola et al. 2021).

**Fig. 1:**
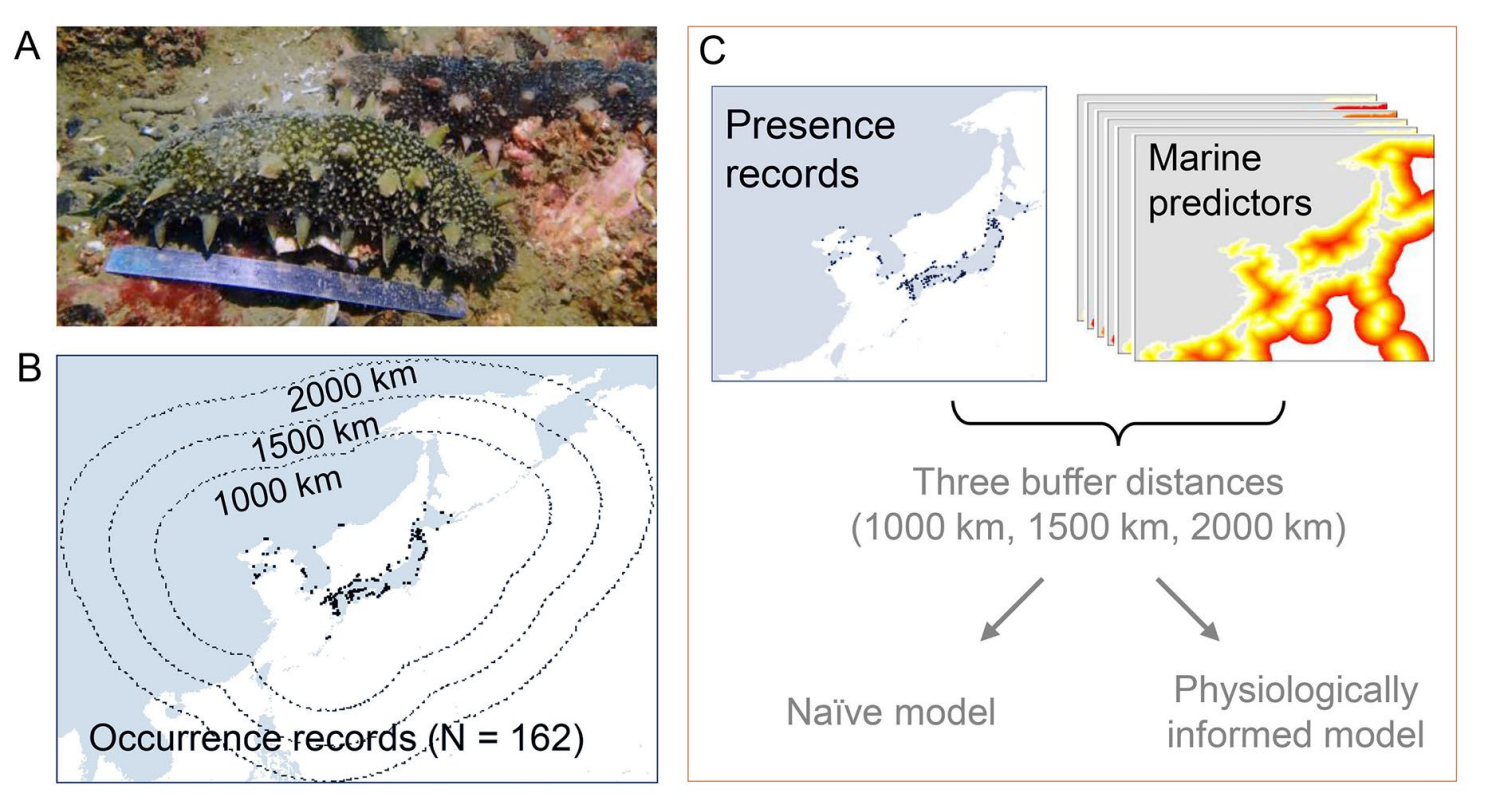
Schematic diagram showing the study design. **A** Photograph of Japanese sea cucumber (*Apostichopus japonicus*), **B** species presence records (black dots) and three distinct buffer regions (equidistant cylindrical projection), and **C** schematic diagram of model design. We calibrated models within three buffer extents (1000 km, 1500 km, and 2000 km), as shown in Fig. 1B. For each calibration extent, we constructed two types of models: physiologically informed and naïve models. The photograph in Fig. 1A was taken on February 6, 2019 in Onagawa Bay (Japan) by Tohoku Ecosystem-Associated Marine Science, and reproduced with permission

The Japanese sea cucumber *Apostichopus japonicus* (Selenka 1867) (Echinodermata: Holothuroidea) (Fig. 1A) is widely distributed along the coastal regions of China, Japan, far eastern Russia, and Korea (Yang et al. 2015). This benthic species is an important aquaculture species of great ecological and economic importance (Yang et al. 2015). In 2019, the aquaculture area of *A. japonicus* in China reached approximately 246,745 hectares, and production of this species was 171,700 tonnes (China Fishery Statistical Yearbook 2020). The production of this species was equivalent to ~ 5.68 billion US dollars at a market price of 33.06 US dollars per kilogram (Yang et al. 2015). This species is particularly sensitive to high temperature and undergoes aestivation—a unique strategy in response to extreme conditions where species stop feeding, exhibit low activity and lose body weight (Yang et al. 2015).

Considering its sensitivity to high temperature regimes, the Japanese sea cucumber represents an ideal model species to explore the importance of integrating physiological information in SDMs. In this study, we developed two sets of SDMs for the Japanese sea cucumber to investigate the potential effects of climate change on habitat suitability for the species: (1) a SDM without considering physiological information (hereafter naïve model); and (2) a SDM that incorporates physiological information as priors to the model (hereafter physiologically informed model). We calibrate models within three buffer extents (1000 km, 1500 km, and 2000 km) and hypothesize that the incorporation of physiological knowledge into SDMs will reduce bias introduced by the choice of calibration areas.

## Materials and methods

### Model calibration area

The Japanese sea cucumber is a coastal water species; thereby, consistent with previous studies (Hu et al. 2021; Stephenson et al. 2020; Zhang et al. 2020), we restricted our analyses within the Exclusive Economic Zone, i.e., waters extending up to 200 nautical miles from shore (Fig. 1B). The extent of the calibration area, a modeling area coherent with the biology and evolutionary history of the target species, is a critical feature to properly calibrate SDMs (Barve et al. 2011). It is always extraordinarily challenging to precisely define the calibration area (VanDerWal et al. 2009; Barve et al. 2011); therefore, following previous studies (e.g., Thuiller et al. 2019; Waldock et al. 2022), we considered the dispersal ability of the Japanese sea cucumber, and delineated calibration areas by creating buffers around species occurrence records (Fig. 1B). Sea cucumbers have planktonic larval stages and exhibit great dispersal potential (Yang et al. 2015). For example, the larvae of the Northern Sea Cucumber *Cucumaria frondosa* were reported to be transported over long distances (ranging from 800 to 1500 km) by ocean currents (So et al. 2011). Interestingly, the adults of sea cucumbers may actively modify their buoyancy and float with ocean currents, up to 90 km per day (Hamel et al. 2019). To reflect the maximal dispersal distance of sea cucumbers, we created buffers with three different radii using equidistant cylindrical projection: 1000 km, 1500 km, and 2000 km. In short, we calibrated both naïve and physiologically informed SDMs within three different buffers (Fig. 1C), and we used these SDMs to project habitat suitability of *A. japonicus* within the largest buffer (i.e., 2000-km buffer).

### Species distribution data and marine predictors

We gathered distribution data for the Japanese sea cucumber through an extensive literature review (Supplementary Table S1). In particular, we treated the three-color variants (red, green, and black) of *A. japonicus* as belonging to the same species (Sun et al. 2010; Zhang et al. 2016). In addition to the literature review, we retrieved distribution data from online repositories including the Global Biodiversity Information Facility (GBIF; https://www.gbif.org) and the Ocean Biodiversity Information System (https://obis.org). Thus, we assembled a total of 458 presence records (Supplementary Fig. S1). Following previous recommendations (Kramer‐Schadt et al. 2013), we filtered presence records by

1. removing records on land.
2. keeping only one presence record per 5 arcminute grid cell (the spatial resolution of marine predictors).
3. performing spatial thinning using a distance of 20 km (Hu et al. 2021) via the *thin* function in R package ‘spThin’ (version 0.2.0; Aiello‐Lammens et al. 2015).

After the above cleaning procedures, we retained 162 presence records for subsequent analyses (Fig. 1B).

To map habitat suitability of the Japanese sea cucumber, we selected six environmental predictors out of an initial suite of 14 candidate predictors (Supplementary Table S2), i.e. water depth, distance to shore, annual mean benthic temperature, annual range of benthic temperature, annual mean benthic salinity, and annual mean benthic current velocity (Supplementary Fig. S2). Empirical evidence supports that these six predictors are ecologically relevant to the geographical distribution of the Japanese sea cucumber. In particular, it is widely accepted that water depth is associated with hydrostatic pressures, and marine benthic species typically will only tolerate certain depth ranges (Brown and Thatje 2014), i.e. less than 200 m for *A. japonicus* (Supplementary Table S3). Therefore, it is reasonable to include water depth into our models. Distance to shore correlates with production pathways and the supply of organic matter from the surface (Lapointe et al. 1994; Miller and Wheeler 2012). Therefore, the two geographical predictors have been commonly used in SDMs for marine organisms, including projecting species redistributions under climate change (e.g., Bosch et al. 2018; Hu et al. 2021; Peters et al. 2022). Temperature and salinity are among the most important environmental factors limiting the distribution of marine organisms (Castro and Huber 2005). Thus, we included annual mean temperature in our analyses instead of maximum temperature because although the Japanese sea cucumber is sensitive to high temperature, it survives high temperatures by aestivating (Wang et al. 2015; Yang et al. 2015). This is supported by the temperature information of species occurrence records: among the 162 records, annual mean temperature of all records was below 26 °C (the upper thermal limit of the Japanese sea cucumber; see Table S3 for details), whereas the maximum temperature of 54.3% (88 out of 162) records was higher than the upper thermal tolerance (Supplementary Fig. S3). We acknowledge that the seasonal variation of sea benthic temperature is large (e.g., Yang et al. 2015). Therefore, apart from annual mean temperature, we further considered the annual range of temperature to reflect the marked seasonal variation. Current velocity may influence the dispersal of marine species (Álvarez-Noriega et al. 2020) and interact with climate warming in driving range shifts (García Molinos et al. 2017). In addition, previous studies pointed out that current velocity may directly affect the movement activity and distribution of *A. japonicus* (e.g., Pan et al. 2015).

Multicollinearity across the six selected predictors was negligible, with variance inflation factor values < 10 (Naimi et al. 2014). We downloaded the two geographical predictors (i.e., water depth and distance to shore) with a spatial resolution of 5 arcminutes from the Global Marine Environment Datasets (http://gmed.auckland.ac.nz) (Basher et al. 2018), and obtained the three marine environmental predictors under average present-day conditions (2000–2014) from the Bio-ORACLE version 2.1 dataset (https://www.bio-oracle.org) (Assis et al. 2018). We assumed that water depth and distance to shore remain constant across time. Future projections of the three marine environmental predictors under four distinct RCP scenarios in 2040–2050 were projected by three atmosphere–ocean general circulation models, including CCSM4, HadGEM2-ES, and MIROC5. We retrieved average outputs of the atmosphere–ocean general circulation models from the Bio-ORACLE version 2.1 dataset (Assis et al. 2018). The raster grids of the two databases did not align, and thus were unified to the same resolution (i.e., 5 arcminutes) using bilinear interpolation.

### Naïve SDMs

In this study, we constructed SDMs via *plateau* approach, which is based on piecewise functions (Brewer et al. 2016; see detailed method in Supplementary Fig. S4). A *plateau* model consists of two intersecting linear functions and an additional constraint on the maximum value. The two linear functions were determined through their intersection point and corresponding slopes, then the maximum value was limited with an additional apex parameter. All parameters in a *plateau* model (see more details in Supplementary Fig. S4) were estimated based on Bayes’ theorem, and numerically implemented via the Markov chain Monte Carlo (MCMC) method (Brewer et al. 2016). The MCMC setting remained identical for all calculations in this study: two parallel chains were run with the same initial values; in each chain a total of 5000 iterations were specified, where 4000 iterations were used for warm-up (also known as burn-in) and the rest 1000 interactions for posterior inference.

As with Feng et al. (2020), we fitted *plateau* models via presence/pseudo-absence data. To achieve this, we randomly generated the same number of pseudo-absences within the calibration areas as the presence data. To avoid the possible effects of pseudo-absence selections on *plateau* models, we developed 25 *plateau* models by randomly generating different pseudo-absence sets within each calibration area. The naïve SDMs were constructed with the default non-informative priors of the marine predictors (Brewer et al. 2016; see more details in Supplementary Fig. S4). Note that we normalized the values of each marine predictor to 0–1, following the recommended practice by Brewer et al. (2016).

### Physiologically informed SDMs

To integrate the physiological information of *A. japonicus* into SDMs, we first collected physiological knowledge from literature of the Japanese sea cucumber related to our marine predictors. As it is commonly the case with marine species, temperature is a parameter for which there is more abundant physiological information on *A*. *japonicus* (Yang et al. 2015). The optimal growth temperature for *A. japonicus* is generally between 15 and 18 °C (Supplementary Table S3). Regarding the upper thermal limit of the Japanese sea cucumber, field surveys reported that the density of this species becomes zero when sea water temperature reaches 24.7 ± 3.2 °C (mean ± standard deviation) (Minami et al. 2019). Furthermore, the threshold temperature inducing aestivation varies, but is normally reported at about 26 °C (Supplementary Table S3). After comprehensively considering the existing evidence, we denote the upper thermal limit of *A. japonicus* as 26 °C. The Japanese sea cucumber is a benthic species living mainly in coastal waters shallower than 40 m, and finds its maximum water depth limit at approximately 200 m (Supplementary Table S3). The species may also tolerate a wide range of salinities (25–45 psu), which covers the whole salinity range within our study region (29–35 psu) (Supplementary Table S3). Accordingly, we did not impose any constraints of salinity into our model. Water current velocity may be expected to exert an effect on both larval dispersal and adult habitat; however, the extent to which this parameter limits the range of *A. japonicus* remains unclear. Therefore, we did not consider any constraints of current velocity in our models. Similarly, no constraints were imposed associated with the distance to the coast as there is no available information about its (likely indirect) effect on this species.

With the given physiological information, we modified the prior distribution of annual mean temperature and water depth (see mathematical expressions in Supplementary Fig. S4). As for annual mean temperature, we constrained the apex value within the range of the optimal temperature, and calculated the slope of the linear function based on the upper thermal limit and apex value. For water depth, we determined the slope of the linear function based on the maximum depth limit (200 m) and optimal depth (0–40 m) (Supplementary Table S3). We normalized each constraint’s values, as we did for each marine predictor (Brewer et al. 2016). We constructed *plateau* models via the R package ‘R2WinBUGS’ (version 2.1–21; Sturtz et al. 2005) that runs the WinBUGS (version 1.4) in batch mode using scripts (Brewer et al. 2016), and performed other analyses, including convergence tests, within R (version 4.0.3) (R Core Team 2020).

### Model performance and projections

We adopted a fivefold spatial block cross-validation approach to evaluate predictive performances of each model. Thus, we split the distribution data into spatial blocks based on rows and columns (10 rows and 10 columns in our case), and randomly allocated these blocks into five folds via the *cv_spatial* function in the R package ‘blockCV’ (version 3.1-3; Valavi et al. 2019). We calibrated models using four folds and validated model performance via the withheld fold. We repeated this procedure until all folds were used for model validation (Zhang et al. 2021 and reference therein). Previous studies highlighted that commonly used discrimination metrics such as AUC (area under the receiver operating characteristic curve) and TSS (true skill statistic) may be misleading when lacking high quality presence-absence data (Leroy et al. 2018; Lobo et al. 2008; Somodi et al. 2017). In our case, we only had species presence data and lacked species’ true absence data (we used randomly generated pseudo-absences as alternatives). Therefore, instead of using AUC or TSS, we measured model performance via the Boyce index using the default setting of *ecospat.boyce* function in the R package ‘ecospat’ (version 3.1; Di Cola et al. 2017), which is specific for presence-only data and is regarded as a more robust metric for this type of data (Hirzel et al. 2006). We further quantified the variable importance for each predictor in the physiologically informed and naïve models determined by randomly permuting the values and then calculating the decrease in the goodness-of-fit measure, called “permutation importance” (Thuiller et al. 2009). Also, we determined the marginal response curves of the two constrained predictors (i.e., water depth and temperature) (Hu et al. 2021). The larvae of the sea cucumber are planktonic and may experience long-distance dispersal by ocean currents (So et al. 2011; Yang et al. 2015). Therefore, we adopted an unlimited dispersal assumption when projecting habitat suitability of the Japanese sea cucumber under future climates, i.e., sea cucumber will be able to inhabit newly suitable ranges in the future (Guisan et al. 2017). We binarized the continuous habitat suitability predictions via a 10% presence probability threshold (Hu et al. 2021; Zhang et al. 2021) and computed the range size changes of the Japanese sea cucumber under future climates.

For the physiologically informed and naïve models, we measured similarity between the suitable ranges predicted by different models via the Jaccard similarity index, which ranges from 0 (completely dissimilar) to 1 (completely similar). Take the models calibrated within 1000 km and 2000 km as an example, first we computed the Jaccard similarity index between all combinations of the suitable ranges predicted by the physiologically informed models (25 predictions of 1000 km buffer model × 25 predictions of 2000 km buffer model = 625 combinations), then conducted the same analyses for the naïve models. With the aid of this prior physiological knowledge, the physiologically informed models should always be able to properly delineate the species’ tolerance of the constrained predictors. Thus, we reasonably hypothesize that the physiologically informed models should be less influenced by the choice of calibration areas (i.e., the choice of pseudo-absences), resulting in more similar spatial distribution patterns (i.e., higher Jaccard similarity index). Also, we explored the range shifts (range expansion and range contraction) of *A. japonicus* under climate change projected by the different models.

## Results

### Model performance and predictor importance

All the models showed high predictive performance with Boyce values over 0.48 (Table 1). Regardless the calibration area, the physiologically informed SDMs showed a slight but insignificant advantage over the corresponding naïve models in terms of Boyce scores (two-sided paired Wilcoxon rank sum test,* p* > 0.05; see Supplementary Fig. S5 for all model predictive indices).

**Table 1:** The Boyce index (mean ± standard deviation) of species distribution models for the Japanese sea cucumber *Apostichopus japonicus*

Buffer (km)	Model type	
	Naïve model	Physiologically informed model
1000	0.519 ± 0.211	0.535 ± 0.178
1500	0.484 ± 0.232	0.537 ± 0.165
2000	0.496 ± 0.254	0.536 ± 0.169

Naïve model refers to the species distribution model without the physiological information, and Informed model is the physiologically informed species distribution model. Buffer refers to the different calibration extents for the species (see Fig. 1)

**Fig. 2:**
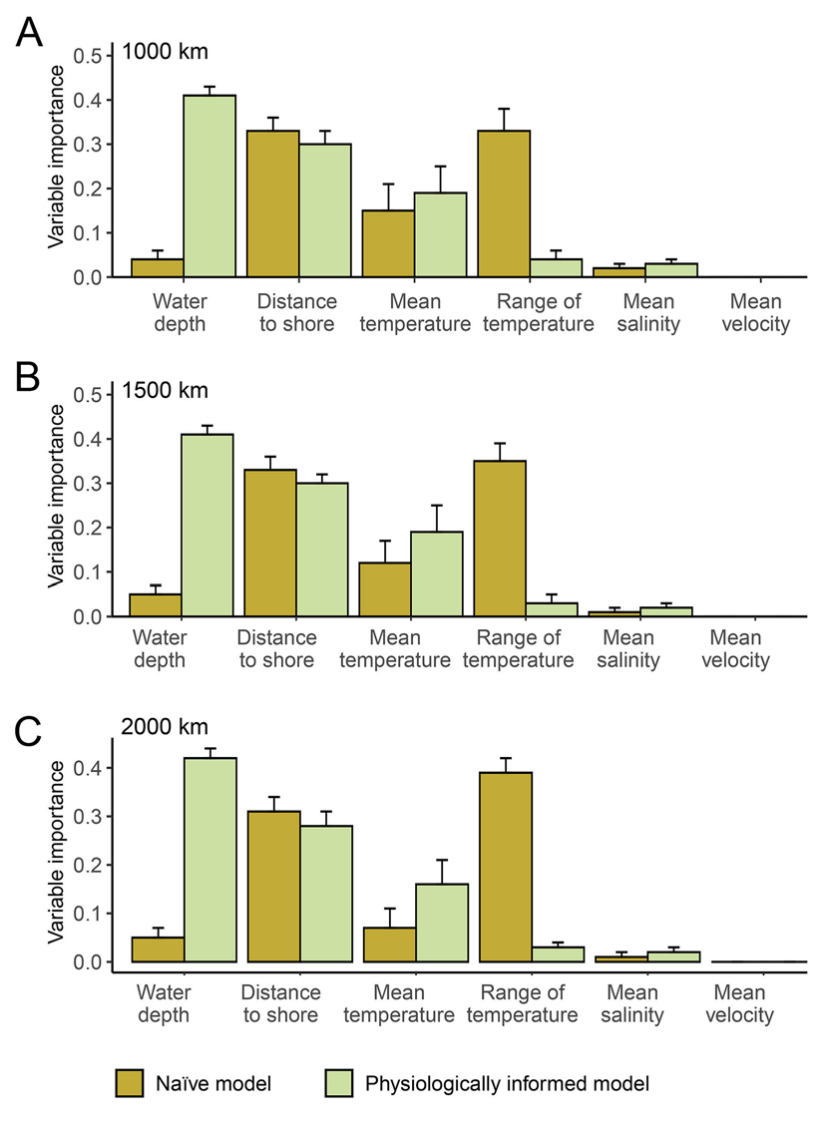
Variable importance in the species distribution models of the Japanese sea cucumber *Apostichopus japonicus* regarding three buffer distances (**A**–**C**) with and without physiological constraints

The physiologically informed and naïve models yielded different results regarding variable importance (Fig. 2). The physiologically informed models showed that water depth, distance to shore, and annual mean temperature represent the three most important predictors explaining the geographical distribution of the Japanese sea cucumber, whereas the naïve models identified the important roles of the distance to shore, annual mean and range of temperature (Fig. 2). All SDMs showed consistently that the contribution of salinity and current velocity was negligible (Fig. 2). The importance of the two constrained predictors (i.e., water depth and annual mean temperature) much higher improved in the physiologically informed models (Fig. 2).

**Fig. 3:**
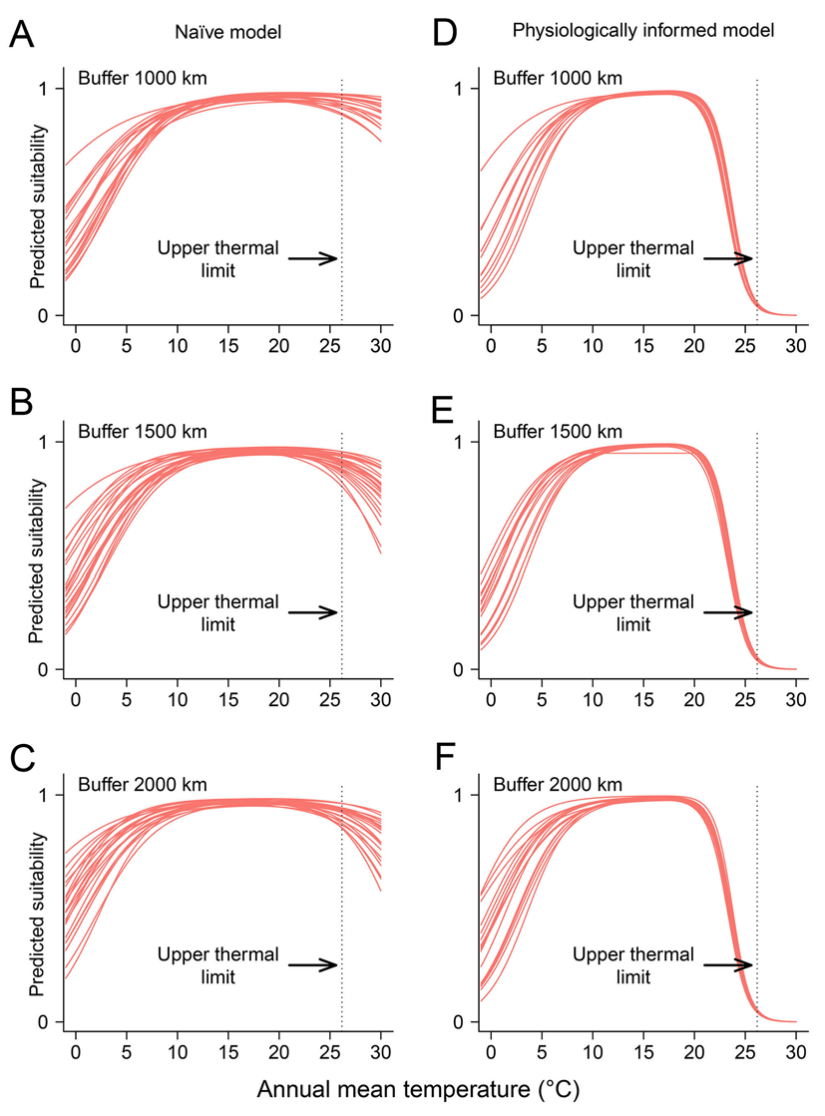
Marginal response curves of the Japanese sea cucumber (*Apostichopus japonicus*) regarding annual mean temperature without (**A**–**C**) and with (**D**–**F**) physiological constraints. Vertical lines represent the upper thermal temperature of the focal species

**Fig. 4:**
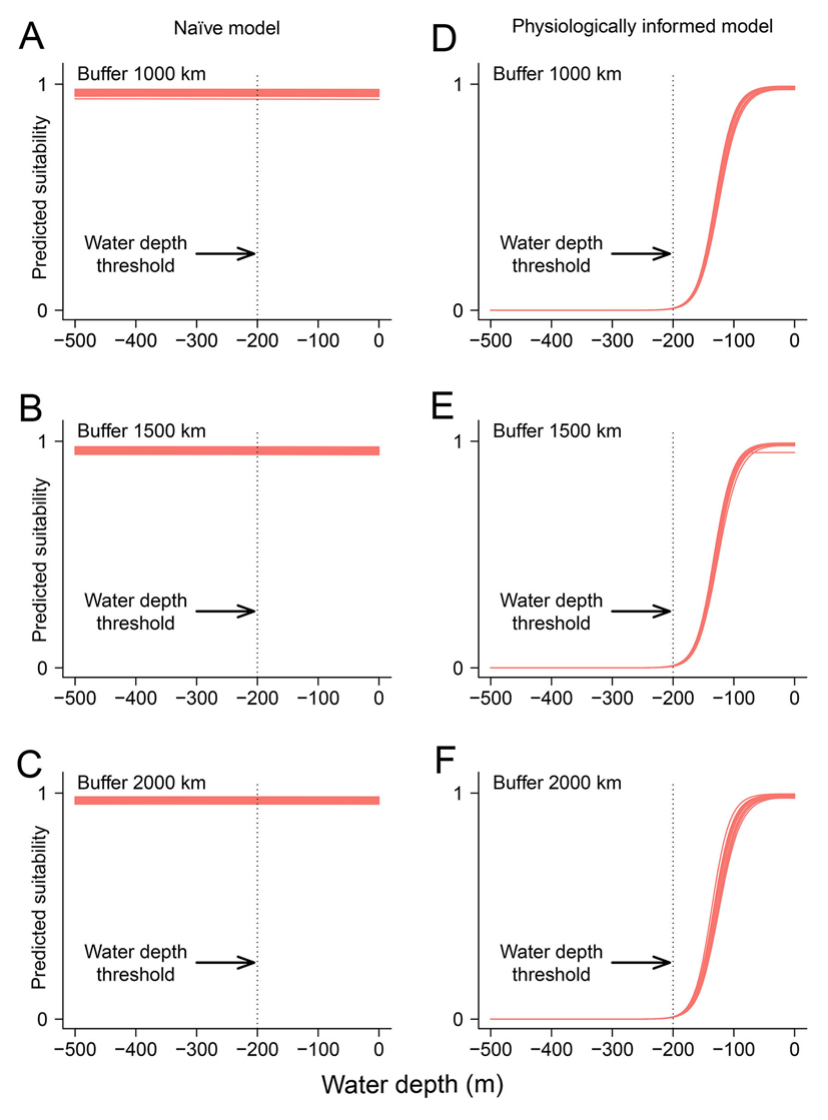
Marginal response curves of the Japanese sea cucumber (*Apostichopus japonicus*) regarding water depth without (**A**–**C**) and with (**D**–**F**) physiological constraints. Vertical lines represent the maximum depth where the focal species can be found

Irrespective of the calibration regions, the two types of models produced different response curves with respect to annual mean temperature and water depth (Figs. 3, 4). On the one hand, the naïve SDMs failed to capture the intolerance of *A. japonicus* to high temperature and deep water with habitat suitability remaining relatively high even for high annual mean temperature (Fig. 3) or water depth (Fig. 4). Conversely, the physiologically informed models successfully detected that high temperature and deep water are unfavorable for the species (Figs. 3, 4). This indicates that the physiologically informed models incorporated successfully the constraints of annual mean temperature and water depth, and these models described better the thermal and bathymetric tolerance of the Japanese sea cucumber.

### Present habitat suitability prediction

**Fig. 5:**
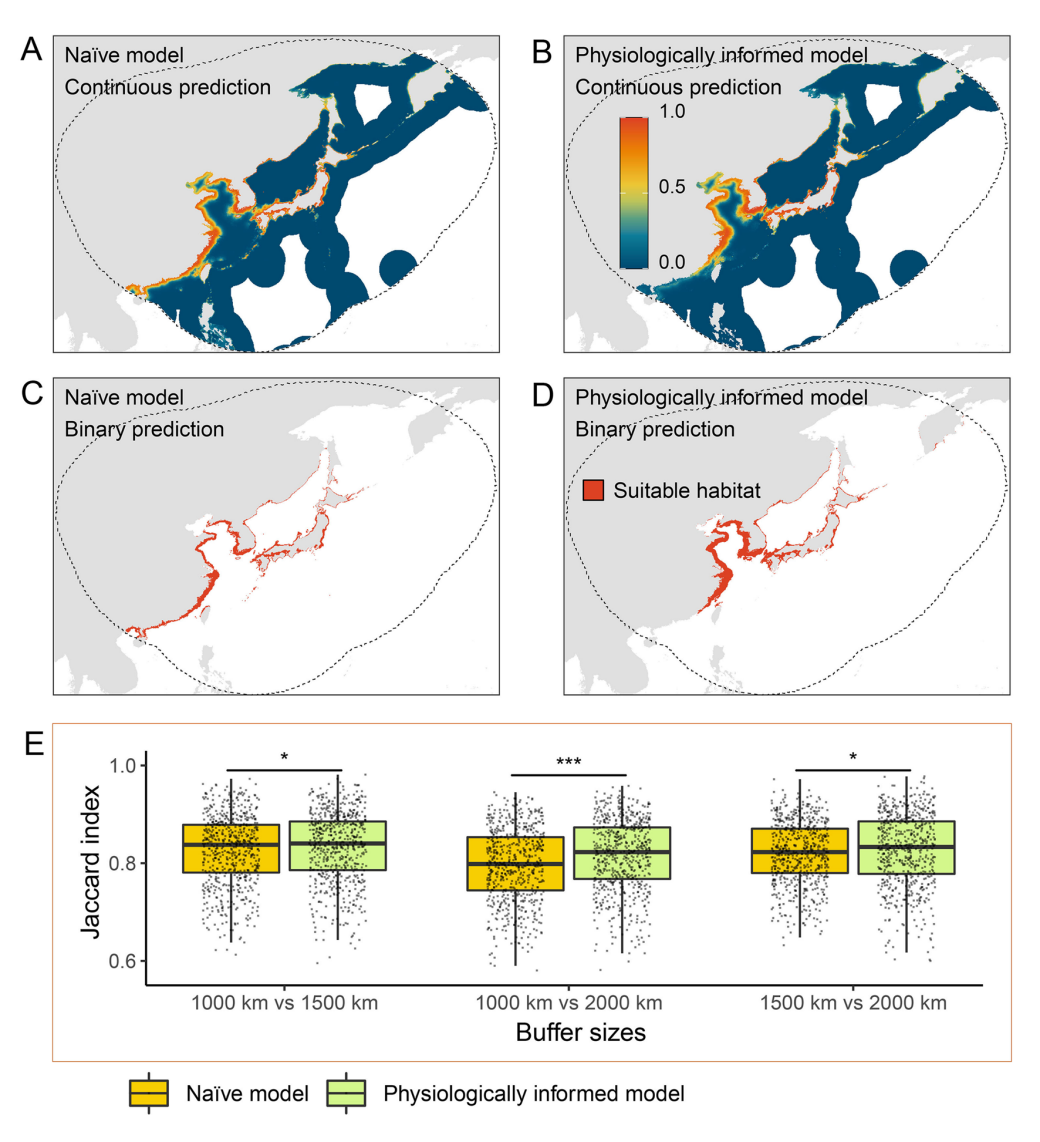
Habitat suitability predictions of the Japanese sea cucumber (*Apostichopus japonicus*) by the two types of species distribution models under present-day climatic conditions using an example of 2000-km buffer. **A**, **B** Continuous habitat suitability predictions (equidistant cylindrical projection). **C**, **D** Binary habitat suitability predictions via a 10% presence probability threshold. **E** Similarity levels between suitable ranges predicted by models calibrated within different buffers. Dashed lines in a–d represent the 2000 km buffer zones. Data in **E** are expressed as mean ± standard error and the asterisk (*) indicates a significant difference (*p* < 0.05) with (***) for *p* < 0.001. **A**–**D** Illustrate one example among 25 models calibrated within 2000 km buffer, habitat suitability predictions for all 25 models and other buffers are available from Figshare (https://figshare.com/s/ec89e8ca525b878fe5fc)

Both models showed consistently that a large proportion of coastal regions of China, Japan, and Korea are suitable for *A. japonicus* under present climatic conditions (Fig. 5). Moreover, suitable ranges for this species are predicted to be available in the south of the Yangtze River estuary in China (Fig. 5). The habitat suitability projected by each model are different in multiple aspects. First, the naïve models projected a more southward distribution limit than the corresponding physiologically informed models (two-sided paired Wilcoxon rank sum test,* p* < 0.05; Supplementary Fig. S6). Second, the physiologically informed models projected significantly larger suitable ranges compared with the naïve models (two-sided paired Wilcoxon rank sum test, *p* < 0.05; Supplementary Fig. S7). Third, and perhaps most importantly, the suitable ranges projected by the physiologically informed models were less influenced by the choice of calibration areas. Our results showed that the Jaccard similarity indices between the suitable ranges predicted by the models fitted to three calibration areas were significantly higher for the physiologically informed models than that for the naïve models (one-sided paired Wilcoxon rank sum test,* p* < 0.05; see Supplementary Table S4 for statistical details), indicating that the physiologically informed models produced more similar predictions under distinct calibration areas (Fig. 5E).

### Climate change effects on future habitat suitability

**Fig. 6:**
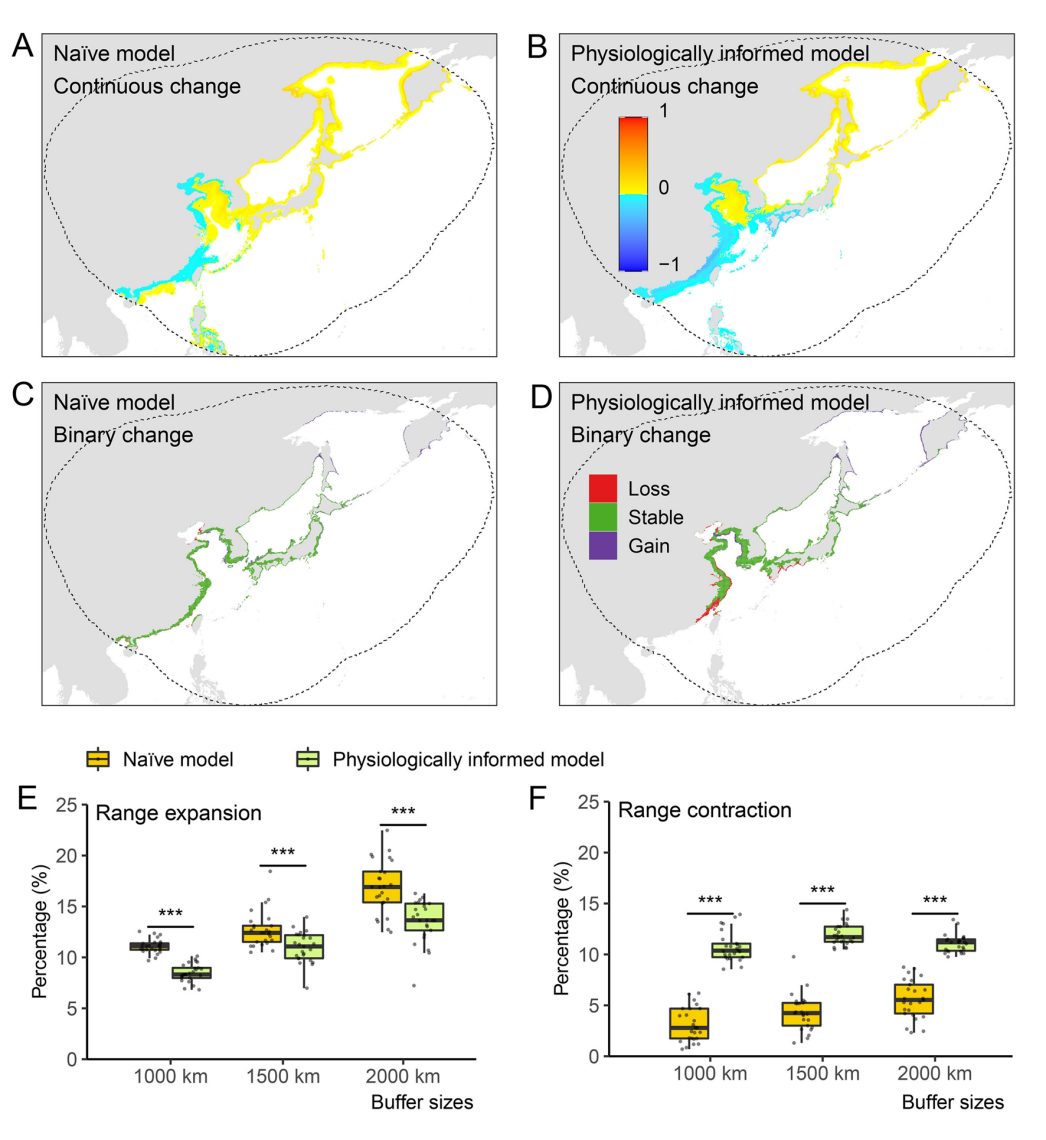
Habitat suitability predictions and changes in projected suitable habitat of the Japanese sea cucumber (*Apostichopus japonicus*) under RCP 8.5 in 2040–2050 by two types of species distribution models using an example of 2000-km buffer. **A, B** Continuous habitat suitability predictions (equidistant cylindrical projection). **C, D** Binary habitat suitability predictions. Range **E** expansion and **F** contraction predicted by models calibrated within different buffers. Dashed lines in a–d represent the 2000 km buffer zones. Data are expressed as mean ± standard error in **E** and **F**, and the asterisk (***) indicates a significant difference (*p* < 0.001). **A**–**D** Illustrate one example among 25 models calibrated within 2000 km buffer. Habitat suitability predictions for all 25 models and other buffers are available from Figshare (https://figshare.com/s/ec89e8ca525b878fe5fc)

The physiologically informed and naïve SDMs projected distinct effects of climate change on the habitat suitability of *A. japonicus* (Fig. 6). We found that the naïve models projected significantly more range expansions but less range contractions under climate changes than the corresponding physiologically informed model (Figs. 6E, F; S8, S9). Taking the models calibrated within 2000-km buffer as an example, under representative concentration pathway (RCP) 8.5 in 2040–2050, compared with the naïve models (range expansion: 16.94% ± 2.59%; range contraction: 5.60% ± 1.89%), the corresponding physiologically informed models projected a significantly lower level of range expansion (13.56% ± 2.08%) (two-sided paired Wilcoxon rank sum test, *V* statistic = 297;* p* < 0.001) but a significantly higher level of range contraction (11.08% ± 0.88%) (two-sided paired Wilcoxon rank sum test, *V* statistic = 0;* p* < 0.001). With respect to range expansion, both types of models suggest that the future suitable ranges of *A. japonicus* will likely expand to northern regions, such as Sakhalin (Fig. 6C, D). As for range contraction, the naïve models identified only evident range contractions in the Bohai Sea, whereas the informed models detected severe range losses in the south Chinese coastal waters, Bohai Sea, Seto Inland, and Ariake seas (Fig. 6C, D).

## Discussion

We used the Japanese sea cucumber as a model to explore the importance of incorporating physiological information into SDMs. Our results highlight that considering physiological information into correlative SDMs may greatly reduce the uncertainties associated with the choice of calibration areas. Compared with the naïve models, our physiologically informed models produced more stable habitat suitability projections with similar model predictive performance. Our findings reaffirm that we should not rely only on predictive performance metrics in SDM studies (Lobo et al. 2008), but rather we should strive to balance statistical and biological considerations and examine the shapes of response curves to avoid any misleading predictions or potentially unreasonable biological trends. Furthermore, our results highlight the significance of integrating species’ physiological knowledge into correlative SDMs, even in the form of a few, simple physiological constraints, as this practice may reduce greatly the uncertainties associated with the choice of model calibration extents, and better inform management and conservation strategies under future climate and environmental changes.

### Critical role of calibration area in SDMs

Among the several critical assumptions of SDMs, a fundamental one is the model ability to capture the meaningful properties of the Hutchinson’s realized niche of species (Guisan and Thuiller 2005; Guisan et al. 2017). This is often implicitly assumed as it is often unclear to what extent this assumption is met according to different research scenarios (Guisan and Thuiller 2005; Guisan et al. 2017), especially the calibration areas (Barbet‐Massin et al. 2010; Guisan et al. 2017; Sánchez‐Fernández et al. 2011; Thuiller et al. 2004). Previous studies demonstrated that SDMs based on regional distribution data, as opposed to whole distribution data, often produce truncated response curves, thus resulting in misleading habitat suitability projections because of extrapolation (e.g., Barbet‐Massin et al. 2010; Sánchez‐Fernández et al. 2011; Thuiller et al. 2004). In our case, although we considered occurrence data from the whole known range of the Japanese sea cucumber, naïve SDMs failed to produce biologically meaningful response curves. Unlike the naïve models, our physiologically informed models were insensitive to the choice of calibration extents in this regard because the prior physiological knowledge had been successfully incorporated as prior information into models. Physiologically informed SDMs have been reported to have different advantages, such as improving extrapolation ability in novel conditions (Feng et al. 2020). Furthermore, our findings suggest that this type of model allows for an easy incorporation of physiological knowledge effectively mitigating the effects of the selection of calibration areas whilst calibrating SDMs. This finding may have broad implications as it is often challenging to correctly define calibration areas in SDM studies because of the frequent lack of data on dispersal potential and historical ranges (e.g., Barve et al. 2011). We encourage future studies to integrate the physiological information of target species to improve model reliability.

### Statistical versus biological importance

In our study, the Boyce index suggests that the predictive capacities of the physiologically informed models were slightly higher than the corresponding naïve models although differences were statistically nonsignificant. Our results are consistent with those of Gamliel et al. (2020), who also reported a marginal performance improvement of physiologically informed models over naïve models. Regarding our results from a statistical point of view, it seems safe to conclude that both physiologically informed and naïve models are reliable given their high Boyce index. However, from a biological point of view, physiologically informed models are more meaningful in that they properly reflected the tolerance of the Japanese sea cucumber to temperature and water depth (Figs. 3, 4). Thus, the question remains whether we should favor biological realism versus blunt measures of statistical performance (Brewer et al. 2016; Mammola et al. 2019b). Previous studies demonstrated that incorporating physiological information into SDMs may make response curves more reasonable (e.g., Feng et al. 2020; Gamliel et al. 2020). Thus far in SDM studies, researchers rely heavily on evaluation criteria, such as AUC, TSS or the Boyce index to justify model reliability (Araújo et al. 2019; Feng et al. 2019; Guisan et al. 2017). We recommend that researchers should treat model predictive performance with caution, and not simply trust high goodness-of-fit values (Santini et al. 2021). In addition to following best-practice standards (Araújo et al. 2019) and reporting necessary information (Feng et al. 2019), these results remind us of the critical role of examining response curves (Zurell et al. 2020).

### Integration of physiological information into SDMs

Physiological information on depth distribution for the species was implemented into our model in terms of its absolute maximum depth limit and a relatively wide range of optimal depth. Without accurate information about the optimal depth range for the species, it is difficult to assess whether this is a sensible choice. The Japanese sea cucumber is associated mainly with shallow water environments, and drastic decreases in abundance have been reported with increases in depth even in very shallow environments. For example, Selin (2001) reported a 830-fold decrease in abundance of the Japanese sea cucumber in the Vostok Bay (Sea of Japan) between 0.5–1.5 m and 8–15 m depth ranges. Therefore, it is also possible that using the absolute maximum depth tolerance limit and a wide range of optimal depth for the species, we may have effectively biased the model towards overoptimistic predictions of the suitable habitat for the species in deeper waters. This highlights the need for the careful assessment of physiological information implemented into models and for the critical interpretation of results. Future studies are required to explore the extent to which incomplete physiological knowledge may bias predictions in this type of physiologically informed models.

Gamliel et al. (2020) reported that physiologically informed models predicted less drastic range shifts under climate change than naïve models. Contrary to the findings of Gamliel et al. (2020), our results show that physiologically informed models predict more severe impacts of climate change with greater range contraction and less range expansion. Nevertheless, the results of Gamliel et al. (2020) and our study indicate consistently that physiologically informed and naïve models projected different future distribution patterns. Incorporating physiological information into SDMs has promising benefits but is still in its infancy; thereby, we encourage future studies to further clarify the vital roles of physiology in predicting species redistribution under climate change.

### Future perspectives

The IUCN expert-based range map shows clearly that the Japanese sea cucumber naturally occurs north of the Yangtze River in China (Hamel and Mercier 2013). This suggests that the Yangtze River outflow may well impose a geographical barrier defining the southern distributional limit of this species; something that seems to be corroborated by the lack of occurrence records for the species from these southern coastal waters. Both the physiologically informed and naïve models predicted a widespread and suitable range for *A. japonicus* south of its known and actual distributional limits (Fig. 6). As any correlative SDMs, physiologically informed models predict where habitat conditions are suitable for species to be present (potential range), not where they actually occur (realized range). Therefore, care is needed when interpreting the output from these models in terms of species ranges particularly when informing those unfamiliar with the methods, such as managers and policy makers. Together with physiology, dispersal capacity, which may be limited by biogeographical barriers as in our present study, species interactions, physical habitat availability (e.g., seafloor substrate composition for benthic species) or human stressors other than climate changes, may all effectively condition the range of a species. There is clearly much to be gained from improving the reliability of SDMs through the combined inclusion of physiological information, species interactions (Zhang et al. 2024) or dispersal capacity and demographic factors (Miller and Holloway 2015). The development of better-resolved predictor layers, both in time and space, is also needed particularly for the marine realm. For example, as a deposit feeder, soft bottom sediments are critical for the habitat of *A. japonicus* (Yang et al. 2015), but high-resolution benthic substrate composition layers are still unavailable.

In summary, we integrated successfully physiological information of the Japanese sea cucumber into SDMs. Our results highlight that physiologically informed SDMs greatly reduce the uncertainties resulting from the choice of calibration extents. Given these promising features, we encourage future SDM studies to incorporate species’ physiological information if available. However, despite their superiority, different types of challenges remain to be addressed, including disentangling the effects of macroclimates and microclimates, the link between field environments and laboratory-derived physiological information, and the lack of developmental stage-specific physiological knowledge [for a detailed discussion on this topic see Feng et al. (2020) and Gamliel et al. (2020)]. Furthermore, because physiological information is costly and time-consuming to acquire, the routine integration of physiology into species distribution modeling may still be a distant reality, especially for multispecies studies.

### Supplementary Information

Below is the link to the electronic supplementary material.

Supplementary file1 (DOCX 2233 KB)

Supplementary file2 (XLSX 23 KB)

## Data Availability

The presence records of the Japanese sea cucumber are available from Figshare (https://figshare.com/s/ec89e8ca525b878fe5fc). Marine predictors can be retrieved from online repositories (https://www.bio-oracle.org and http://gmed.auckland.ac.nz). The present-day and future habitat suitability predictions of the Japanese sea cucumber are available from Figshare (https://figshare.com/s/ec89e8ca525b878fe5fc).
