# Supplementary material for: Incorporating physiological knowledge into correlative species distribution models minimizes bias introduced by the choice of calibration area
Source: Mar Life Sci Technol. 2024 May 13;6(2):349–62. doi: 10.1007/s42995-024-00226-0 (PMC11136901; doi:10.1007/s42995-024-00226-0)
Supplement: Supplementary file 1 — Supplementary file1 (DOCX 2233 KB) [file 42995_2024_226_MOESM1_ESM.docx]

**Supporting Information for:**

**Incorporating physiological knowledge into correlative species distribution models minimizes bias introduced by the choice of calibration area**

## Figure S1


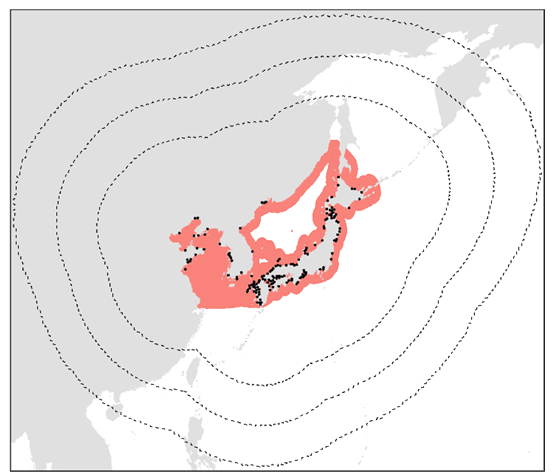


**Figure S1.** Presence records of the Japanese sea cucumber *Apostichopus japonicus* collected from literature and online repositories (equidistant cylindrical projection). The red shaded region represents natural range of the Japanese sea cucumber *Apostichopus japonicus* from IUCN. Dashed lines represent the boundary of three buffers.

## Figure S2


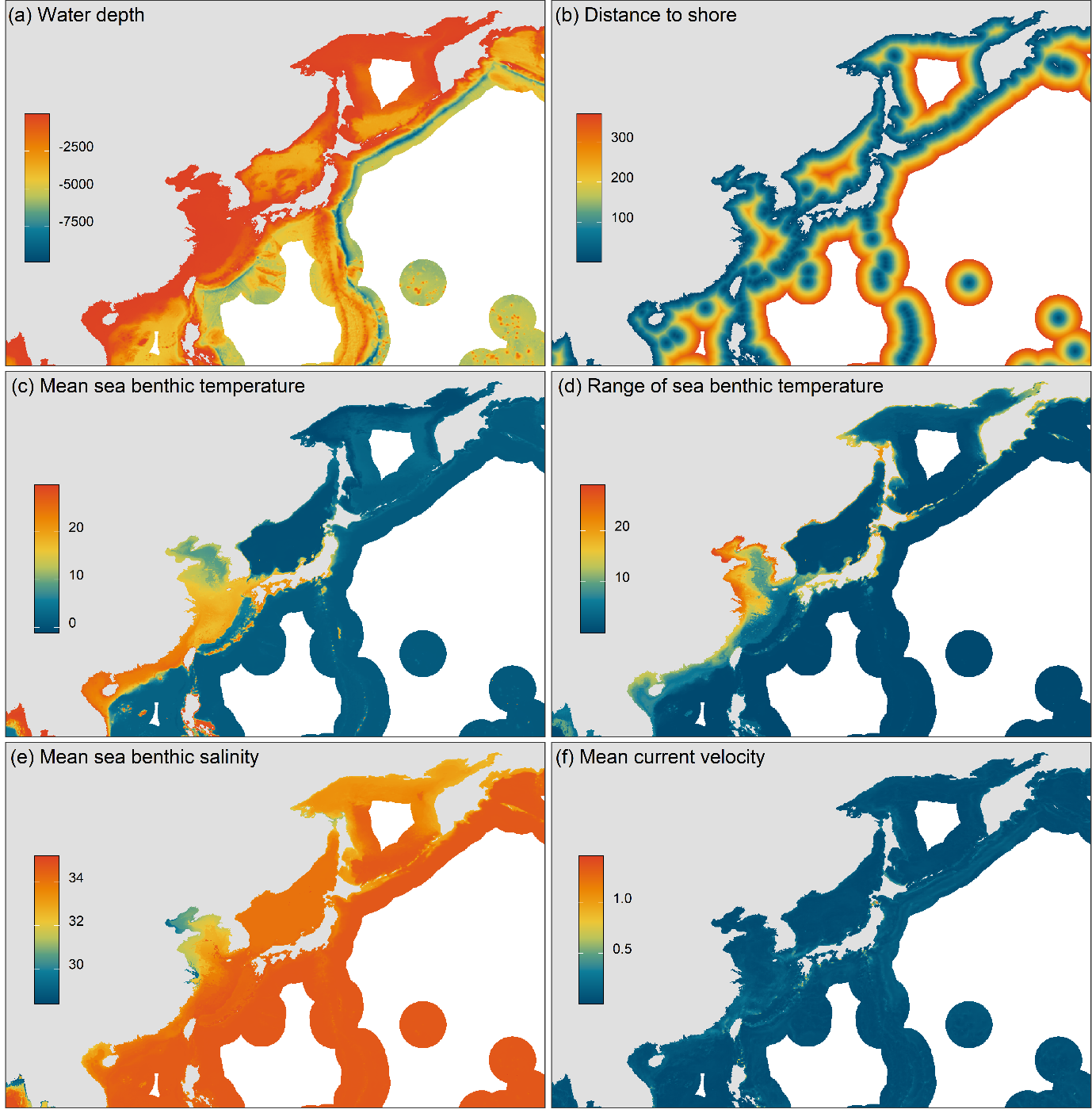


**Figure S2.** The six marine predictors used in the analyses (equidistant cylindrical projection).

## Figure S3


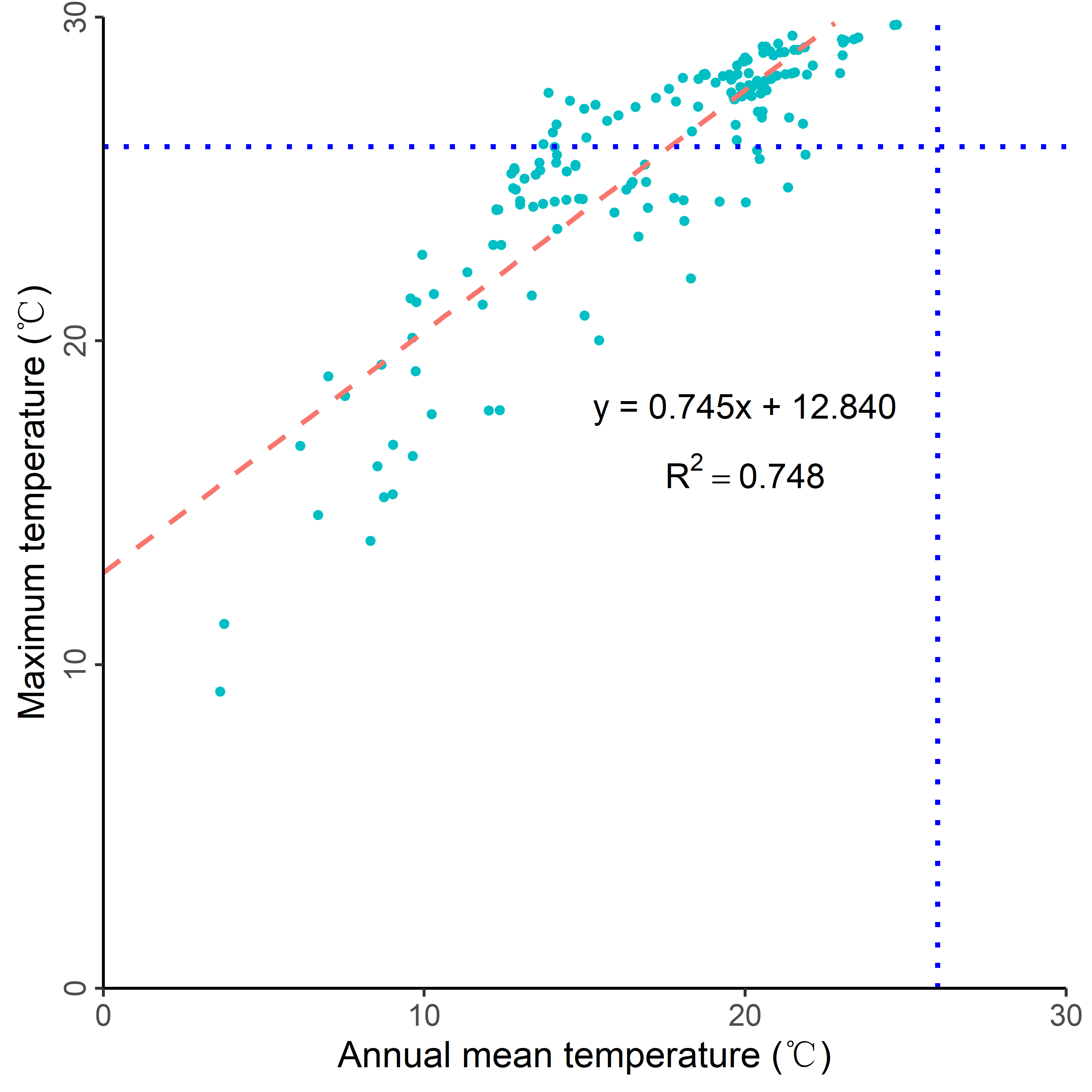


**Figure S3.** The correlation between annual mean temperature and maximum temperature of species occurrence records. We extracted temperature information of 162 occurrence records of Japanese sea cucumber, and found that annual mean temperature and maximum temperature are highly correlated (Pearson correlation coefficient = 0.866, *p* < 0.01). The vertical and horizonal blue dashed lines represent the upper thermal limit (26 °C) of Japanese sea cucumber.

## Figure S4


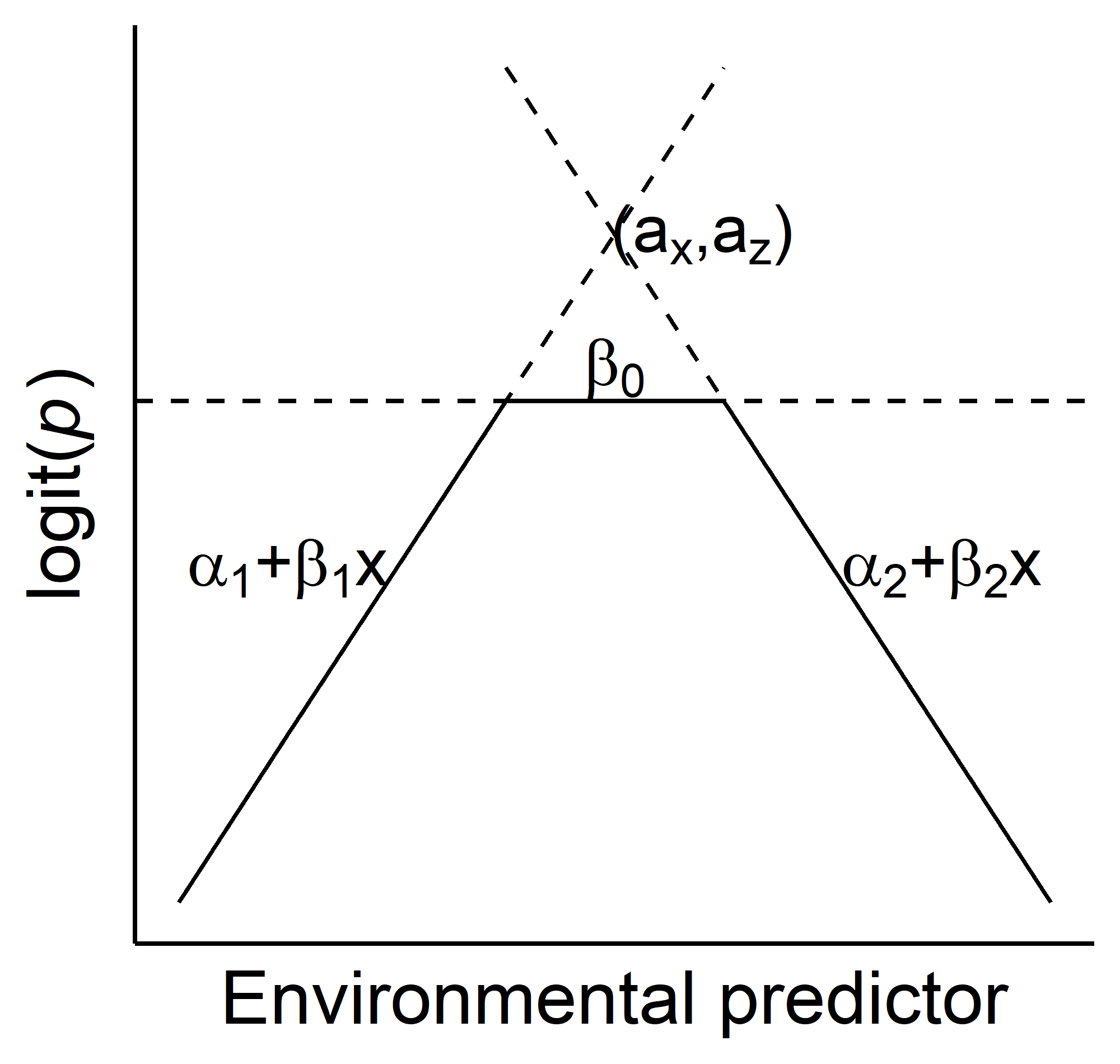


**Figure S4**. The relationships between environmental predictor and presence probability through a logit link function. The piecewise function is illustrated by three segments in solid line with the intersection point (*a_x_, a_z_*). This figure was adopted from Figure 1a in Brewer et al. 2016.

In the multivariate case, the conceptual *plateau* model can be expressed as follows:

$$y_{n} \sim\mathrm{Bernoulli}\left( p_{n} \right)$$

$$\mathrm{logit}\left( p_{n} \right)=\min\left( z_{n},\beta_{0} \right)$$

$$\left( z_{n}-a_{z} \right)^{2}=\sum_{i=1}^{m} \beta_{i,1}\left( x_{n,i}-a_{x_{i}} \right)^{2}I[x_{n,i}<a_{x_{i}}]+\beta_{i,2}\left( x_{n,i}-a_{x_{i}} \right)^{2}I[x_{n,i}\geq a_{x_{i}}]+\sum_{i>j} \gamma_{i,j}\left( x_{n,i}-a_{x_{i}} \right)\left( x_{n,j}-a_{x_{j}} \right)$$

for $\beta_{i,1}>0$ and $\beta_{i,2}<0$

where *n* is the record from the species’ distribution data, *m* is the number of environmental predictors, *y_n_* is the dichotomous response of presence (*y_n_* = 1) or absence (*y_n_* = 0), and *p_n_* is the probability of presence. *p_n_* is calculated through a logit link function, which is widely adopted for binary data (Cramer 2003). In the *plateau* model, the maximum value (*z_n_*) based on two linear functions is constrained by $\beta_{0}$, and the calculation toward *z_n_* considers the interactions among predictors (γ). Parameter $\beta_{m,1}$ and $\beta_{m,2}$ are the slopes of two linear functions, and point (*a_x_*, *a_z_*) is the intersection point of two functions. *x* is the value of the predictor.

Physiological knowledge of marine predictors can be included in *plateau* model via the following formulas:

$$a_{x_{i}}\sim N\left( 0.5,1 \right) I\left[ -1,2 \right] \forall i=2, 3, 4,5$$

$a_{x_{1}}\sim U\left( -200, -40 \right)$ ##

$$a_{z}\sim N(0, 10)$$

$$\beta_{i,1}\sim N\left( 0, 100 \right) I\left[ 0,\infty\right] \forall i=2, 4, 5$$

$\beta_{1,1}=\left( \frac{a_{z}-logit(0.02)}{a_{1}-(-200)} \right)^{2}$ ##

$\beta_{3,1}=\left( \frac{a_{z}-logit(0.02)}{a_{3}-12.30} \right)^{2}$ ##

$$\beta_{i,2}\sim N\left( 0,100 \right) I\left[ 0,\infty\right] \forall i=1, 2, 4, 5$$

$\beta_{3,2}=\left( \frac{a_{z}-logit(0.02)}{a_{3}-26.02} \right)^{2}$ ##

$$\beta_{0}=a_{z}-\exp\{\beta_{0}^{*}\}$$

$$\beta_{0}^{*}\sim N(0, 10)$$

$$\gamma_{i,j}\sim U\left( 0, 1 \right) \forall i<j$$

where *i* refers to each predictor, with 1 = water depth, 2 = distance to shore, 3 = temperature, 4 = salinity, and 5 = current velocity. $\beta_{0}$ is indirectly calculated by $\beta_{0}^{*}$.

## Please note that all constraints need to be normalized during model fitting.

Cramer, J. S. (2003). Logit models from economics and other fields. Cambridge University Press.

## Figure S5


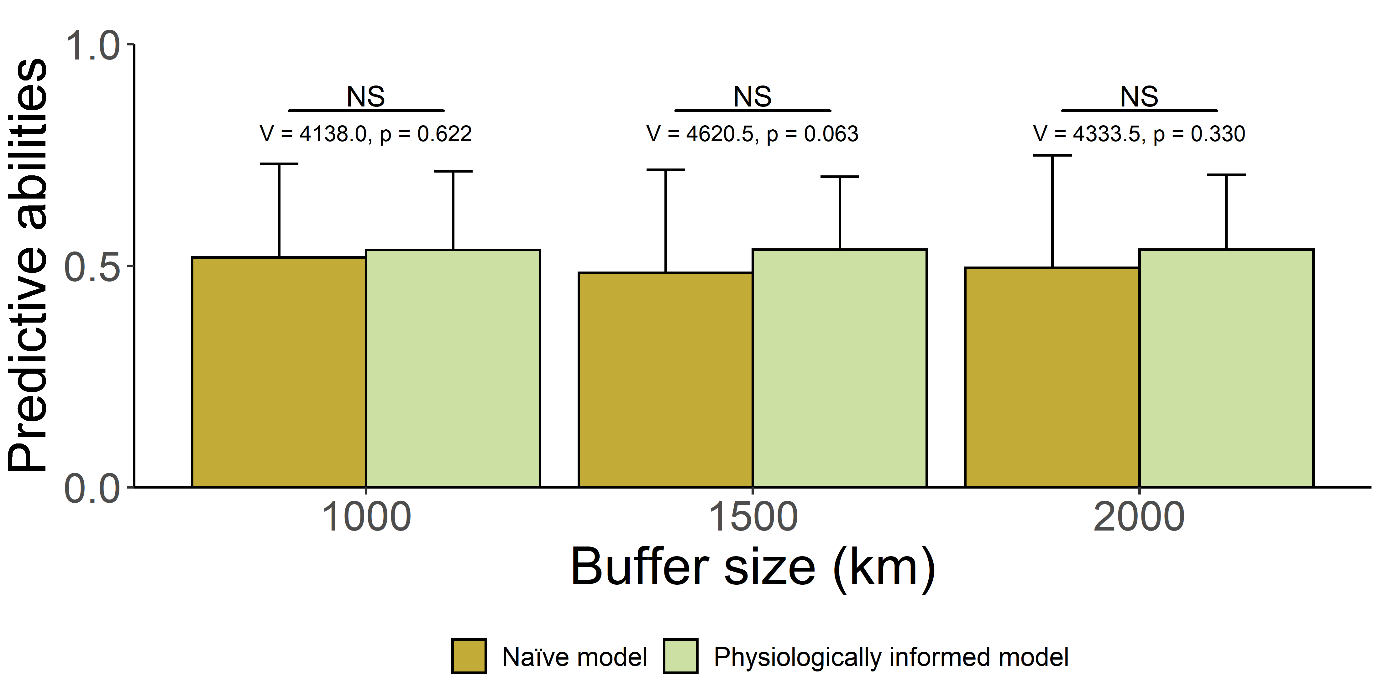


**Figure S5**. Statistical significance test of model predictive abilities (Boyce) between naïve and physiological informed models. We performed two-sided paired Wilcoxon rank sum test. NS stands for not significant difference (*p* > 0.05).

## Figure S6


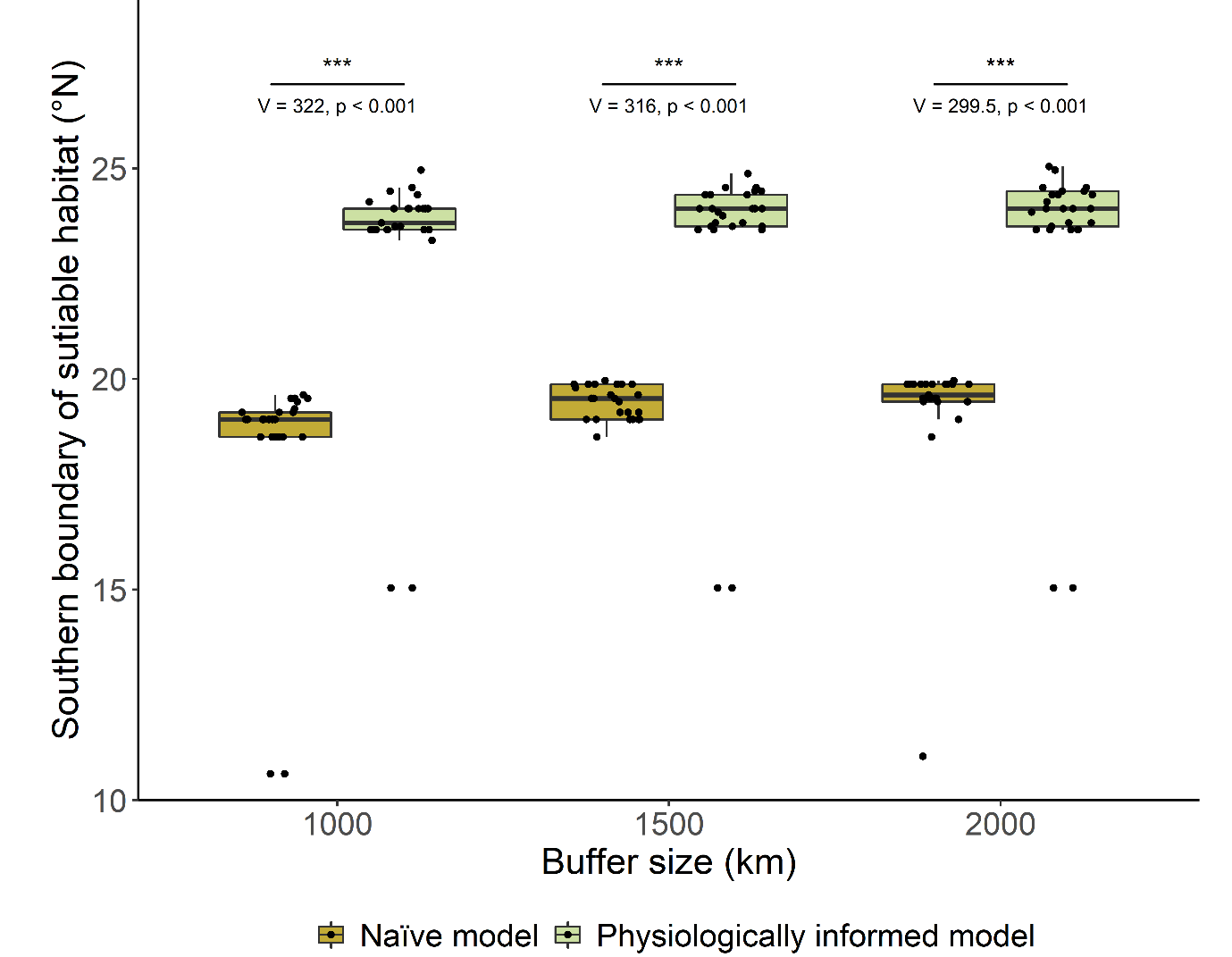


**Figure S6**. The predicted southern boundary of suitable habitat, which is presented by the latitude of the southern boundary, of Japanese sea cucumber by naïve and physiologically informed models. The asterisk (***) indicates a significant difference (*p* < 0.001).

## Figure S7


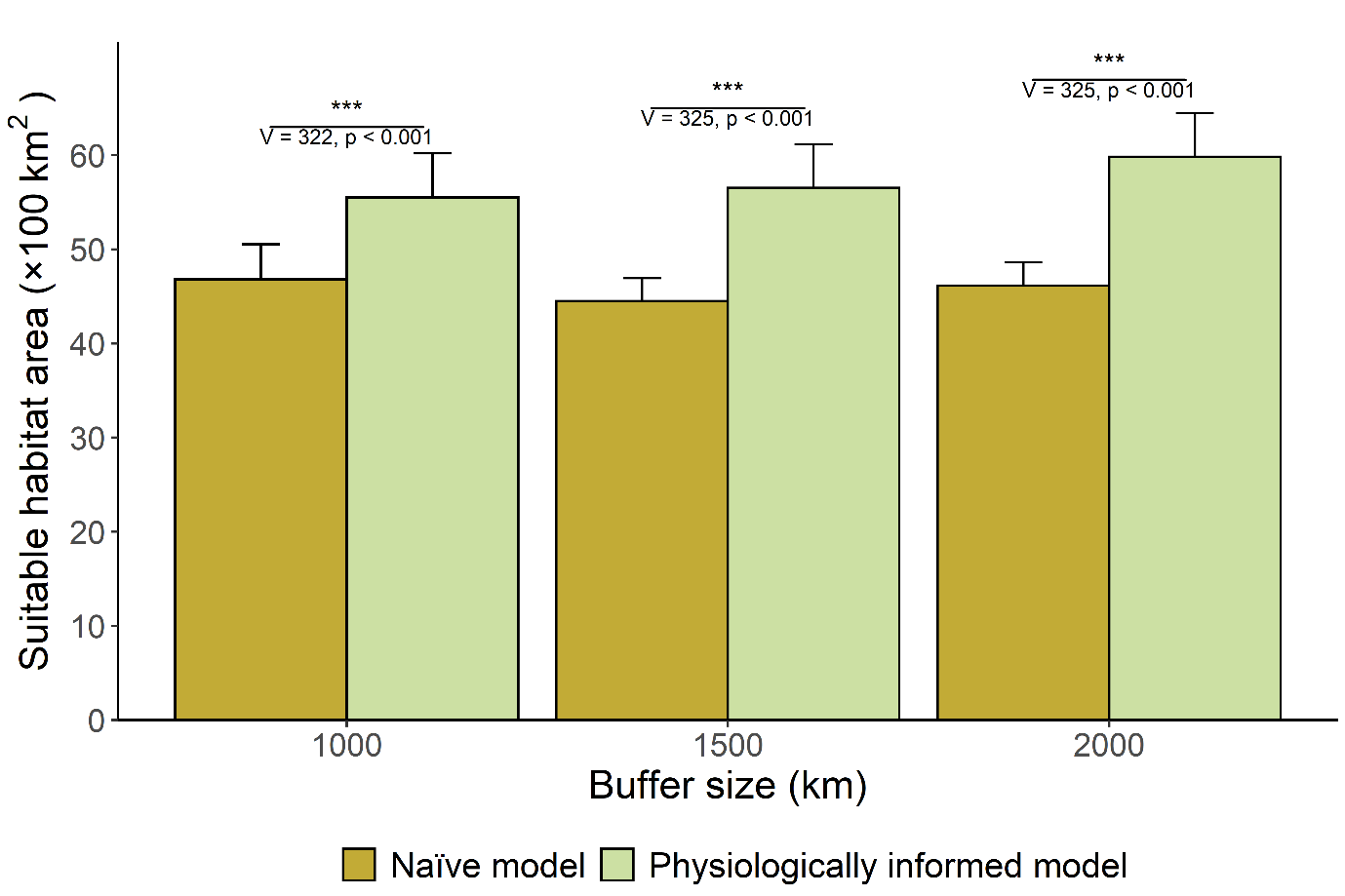


**Figure S7**. The predicted suitable ranges of Japanese sea cucumber by naïve and physiologically informed models. We compared the differences in suitable ranges between two models using two-sided paired Wilcoxon rank sum test. The asterisk (***) indicates a significant difference (*p* < 0.001).

## Figure S8


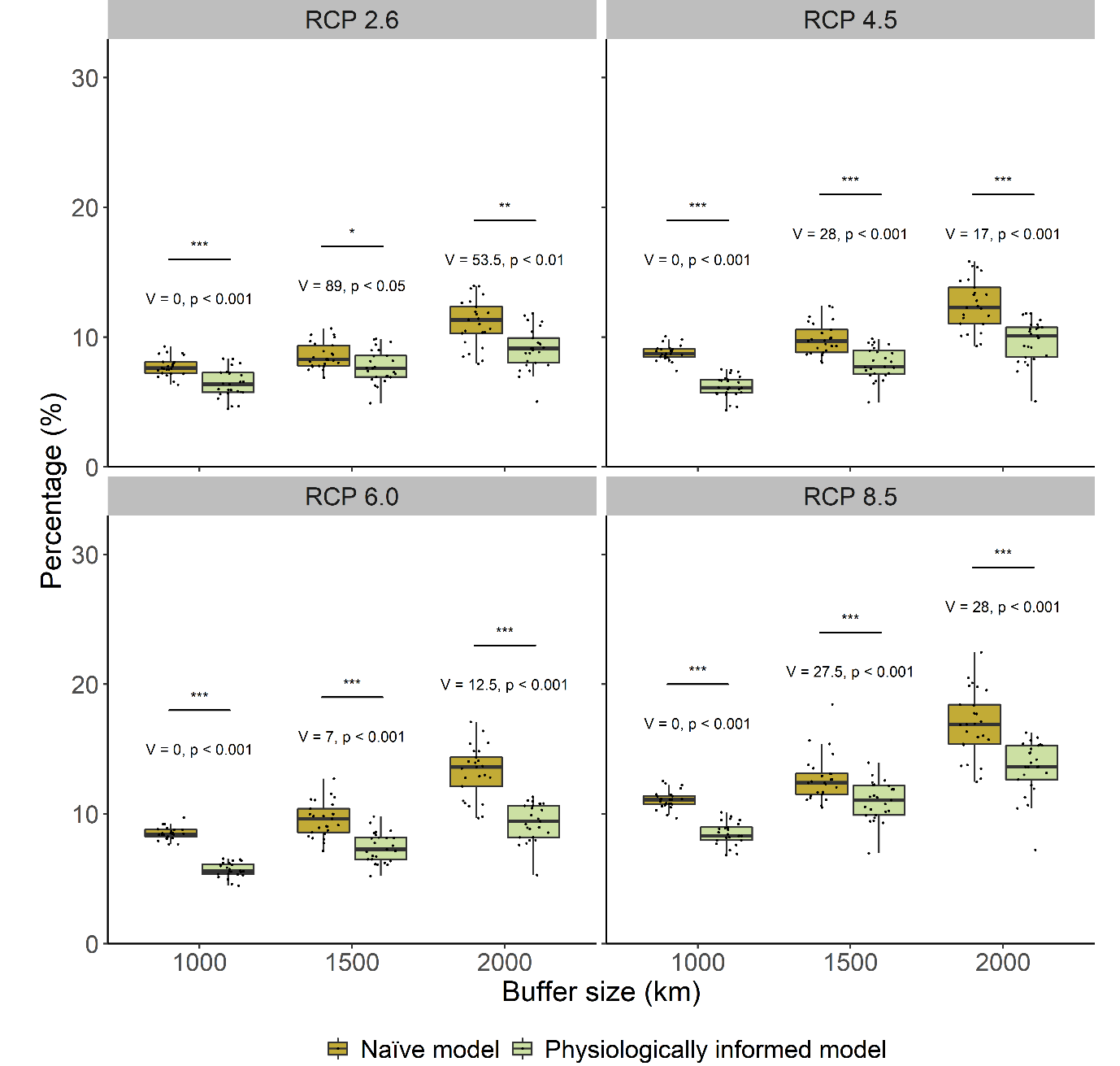


**Figure S8**. The predicted range expansion of Japanese sea cucumber by naïve and physiologically informed models. We performed two-sided paired Wilcoxon rank sum test. * indicates *p* < 0.05, ** indicates *p* < 0.01, and *** indicates *p* < 0.001.

## Figure S9


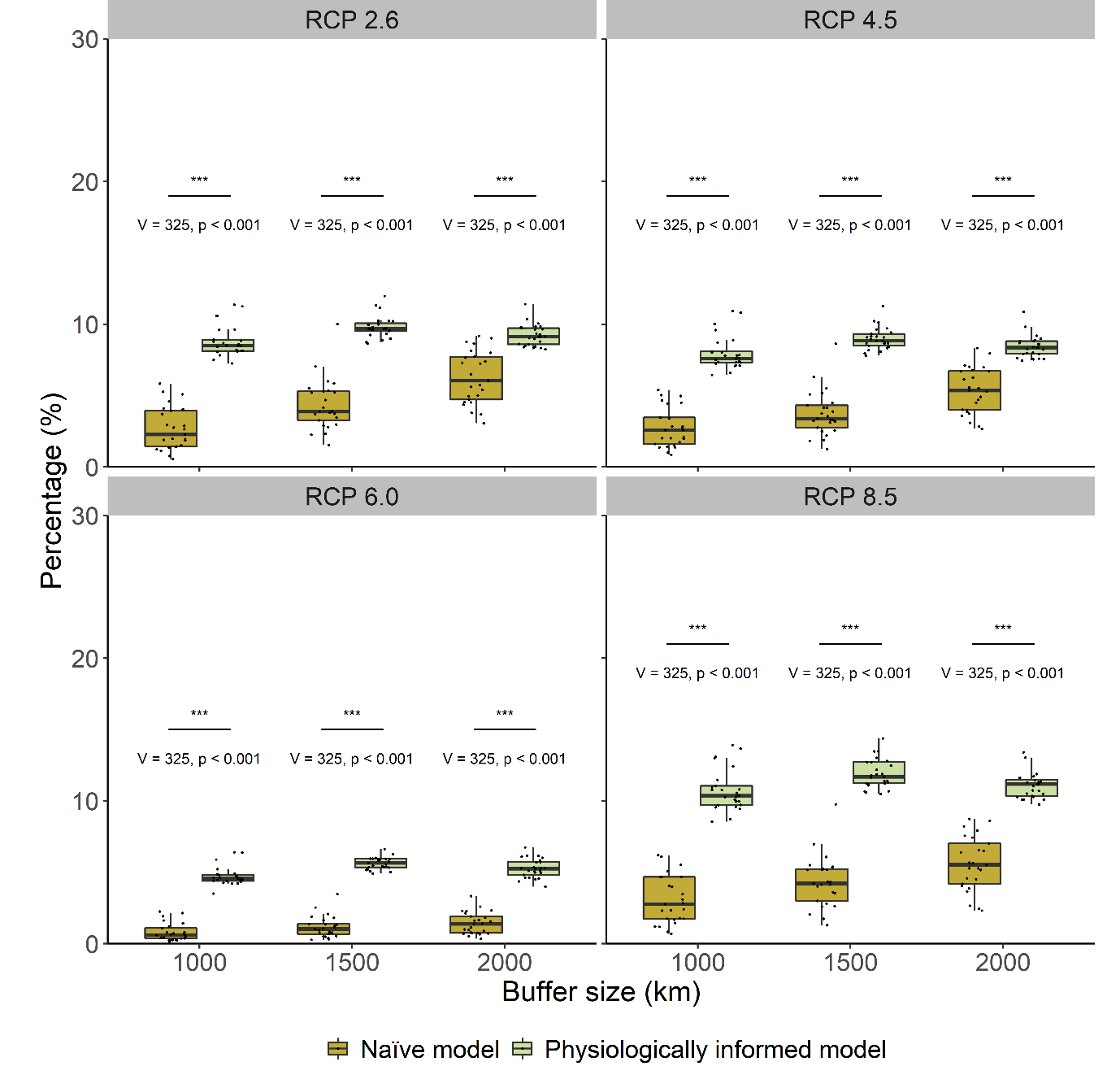


**Figure S9**. The predicted range contraction of Japanese sea cucumber by naïve and physiologically informed models. We performed two-sided paired Wilcoxon rank sum test. The asterisk (***) indicates a significant difference (*p* < 0.001).
